# P-1103. “Take 5”: Positive Reinforcement to Improve Hand Hygiene Rates

**DOI:** 10.1093/ofid/ofaf695.1298

**Published:** 2026-01-11

**Authors:** Vanessa Kung, Adrian Clifford, Lynette Hathaway, Mary Jo Zubaty, Carli Viola-Luqa, Shelia CloudWoods, Joseph Penzelik, Jody Feigel, Elise M Martin

**Affiliations:** VA Pittsburgh Healthcare System, Pittsburgh, PA; VA Pittsburgh, Pittsburgh, Pennsylvania; VA Pittsburgh, Pittsburgh, Pennsylvania; VA Pittsburgh, Pittsburgh, Pennsylvania; VA Pittsburgh, Pittsburgh, Pennsylvania; VA Pittsburgh, Pittsburgh, Pennsylvania; VA Pittsburgh, Pittsburgh, Pennsylvania; VA Pittsburgh, Pittsburgh, Pennsylvania; VA Pittsburgh Healthcare System, Pittsburgh, PA

## Abstract

**Background:**

Consistent hand hygiene compliance is a crucial component of infection prevention. Although incentives programs to promote hand hygiene compliance in healthcare settings can improve patient safety and reduce healthcare-associated infections, limited data exist on non-monetary rewards.

**Figure:**
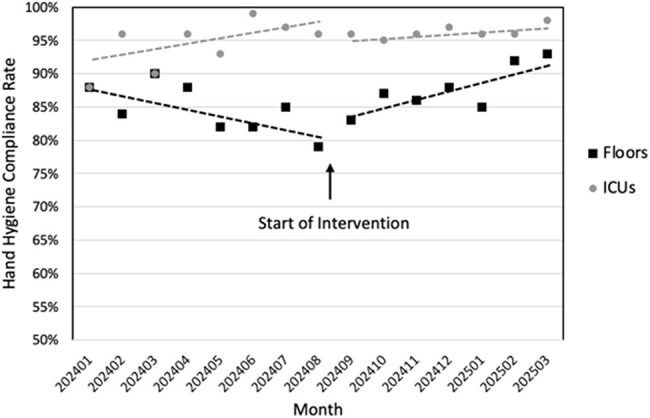

**Methods:**

We implemented a positive reinforcement hand hygiene program in a large VA hospital in 8/2024, and assessed its impact on hand hygiene compliance. The program incorporated hand hygiene education, code word ("take 5") education, a tiered non-monetary incentive (stickers, premium parking spot, and/or lunch with leadership) points program, real-time feedback (verbal and coupons), and performance tracking by unit. Healthcare personnel were given one point for performing hand hygiene or wearing personal protective equipment (PPE), or 2 points for using the non-confrontational code word "take 5" to remind coworkers to perform hand hygiene or wear PPE. Individual staff winners with the most points were selected every 2 weeks. Hand hygiene compliance rates were monitored through direct observation by trained staff (unit based and secret shoppers), pre- (1/2024-7/2024) and post- (10/2024-3/2025) intervention, and feedback was provided to units and individual staff regularly. Dates 8/2024-9/2024 were excluded during education roll out.

**Results:**

The hand hygiene compliance rate on medical/surgical floors was decreasing pre-intervention, and the baseline rate in the 3 months prior to intervention was 83%. Compliance increased post-intervention (p = 0.0321), with current average 89%. The average hand hygiene compliance rate in ICUs was stable pre- and post-intervention, at 94% and 96%, respectively. An average of 208 "take 5" observations were submitted per month, with an average of 11 observations per month of non-infection prevention employees using the "take 5" code word to remind their colleagues to perform hand hygiene or wear PPE. 17 Staff winners were identified, and most elected the premium parking spot.

**Conclusion:**

Preliminary results suggest that a positive reinforcement hand hygiene campaign, including code words and non-monetary rewards, can be effectively adopted in a large healthcare facility to increase hand hygiene compliance.

**Disclosures:**

All Authors: No reported disclosures

